# Prescribing patterns of polypharmacy in Korean pediatric patients

**DOI:** 10.1371/journal.pone.0222781

**Published:** 2019-10-01

**Authors:** Soo-Min Jeon, Susan Park, Sandy Jeong Rhie, Jin-Won Kwon

**Affiliations:** 1 College of Pharmacy and Research Institute of Pharmaceutical Sciences, Kyungpook National University, Daegu, Korea; 2 College of Pharmacy and Division of Life and Pharmaceutical Sciences, Ewha Womans University, Seoul, Korea; University of Toronto, CANADA

## Abstract

**Background:**

Several studies have examined the risk and health outcomes related to polypharmacy among the elderly. However, information regarding polypharmacy among pediatric patients is lacking.

**Objective:**

The aim of this study was to investigate the prevalence of polypharmacy and its related factors among the pediatric population of South Korea.

**Methods:**

We used national claim data from the Health Insurance Review and Assessment Service—Pediatric Patients Sample (HIRA-PPS) in Korea originating from 2012 through 2016. Polypharmacy was defined as a daily average of two or more drugs used yearly. Complex chronic conditions (CCCs) were examined to evaluate concomitant chronic diseases in pediatric patients. Age-specific contraindications and potential drug-drug interactions were assessed according to criteria established by the Korea Institute of Drug Safety & Risk Management (KIDS). Descriptive statistics and logistic regression were conducted to analyze the status of polypharmacy and its associated risk factors in pediatric patients.

**Results:**

The 5-year prevalence of pediatric polypharmacy in pediatric patients was 3.7%. The prevalence of polypharmacy was much higher in younger pediatric patients: 9.5% for patients between the ages of 1–7 years, 0.9% for ages 6–11 years, and 1.1% for ages 12–19 years. Pediatric patients with CCCs, Medical Aid benefits, or a hospital admission history had a significantly higher prevalence of polypharmacy when compared to their counterparts without those conditions. The most commonly prescribed drugs were respiratory agents (29%) followed by anti-allergic drugs (18.7%), central nervous system agents (15.9%), antibiotics (10.1%), and gastrointestinal drugs (7.7%). There was a positive correlation between the daily average number of inappropriate prescriptions and the degree of polypharmacy, especially in pediatric patients between the ages of 1–7 years. Contraindications and potential drug-drug interactions occurred in 11.0% and 10.1% of patients exposed to polypharmacy, respectively.

**Conclusions:**

One in ten pediatric patients under the age of 7 years was prescribed two or more concurrent drugs on average per day. Furthermore, pediatric patients exposed to polypharmacy showed an increased risk of inappropriate drug use. The implementation of a medication review system that considers pediatric patient polypharmacy exposure would reduce inappropriate drug use and prevent unwanted adverse outcomes.

## Introduction

Polypharmacy is a common global phenomenon caused by increasing comorbidities [1, 2], and it increases the risk of inappropriate medicine use, drug-drug interactions, and adverse health outcomes [3–5]. Several polypharmacy studies have been conducted in many countries and most examine elderly populations [2]. Their results indicate that there is an association between polypharmacy and adverse health outcomes such as falls, hospitalization, and mortality [4, 5]. Concerns about polypharmacy in pediatric patients are on the rise [6] because of the increasing number of prescriptions used to treat chronic conditions in pediatric patients [7–9].

Very few studies are available regarding polypharmacy among pediatric patients, and they are limited to a single region (i.e. western countries) or a single drug class (i.e. antipsychotics) [10, 11]. Several studies have investigated polypharmacy exposure for a wide variety of medicines and their related outcomes based on population-level data [12, 13]. One study examining pediatric patients from 463 hospitals in the United States (U.S.) found that 10% of children less than one year of age received 11 medications on the first day of admission into a healthcare facility and were cumulatively exposed to 29 medications during 7 days of admission [12]. Another U.S. study by Feinstein et al. examining polypharmacy among outpatients reported that 35% of pediatric patients (N = 232,240) were exposed to more than two concurrent drugs for at least one day during the one-year observation period [13]. It also showed that the potential for drug-drug interactions increased as the number of drugs being prescribed increased [13]. However, there is a lack of information on pediatric polypharmacy for many drug classes in East Asia countries.

Polypharmacy is generally characterized as the use of multiple medications. However, accurately defining pediatric polypharmacy and quantify related exposures is challenging [14]. Some studies have defined polypharmacy based on the actual number of prescribed drugs [12, 13] while other studies have assessed polypharmacy using the average number of drugs prescribed [15, 16]. The method that uses the maximum number of concurrent drugs is sensitive when multiple drugs are used to treat acute illnesses; however, the method that uses an average number of drugs is useful for assessing long-term exposure to medicines used daily for the treatment of chronic conditions. As chronic diseases in pediatric patients increases, along with a tendency toward polypharmacy, the duration of drug exposure and the number of active ingredients in the drugs being prescribed should be carefully considered [17].

The objectives of this study were to investigate the prevalence of polypharmacy based on the daily average number of medications taken and to assess the risk factors associated with polypharmacy in pediatric patients in South Korea.

## Materials and methods

### Data source

We used the Health Insurance Review and Assessment Service–Pediatric Patients Sample (HIRA-PPS) claim database using data originating from 2012 through 2016. South Korea has a universal health coverage system consisting of the National Health Insurance Service (NHIS) and the Medical Aid Program, which covers approximately 97% and 3% of the Korean population, respectively. The HIRA manages and assesses claim data from NHIS and Medical Aid for 46 million patients (about 90% of the total population that visits medical institutions annually) that need to be reimbursed from approximately 80,000 health care centers per year. Data from 10% of all patients under the age of 20 were selected from the HIRA-PPS database using a random sampling of sex and age strata [18]. The HIRA-PPS also contains patient socio-demographic characteristics, medical treatment, prescription drug information, and disease diagnoses based on the International Classification of Diseases, 10th revision (ICD-10). We included patients only between the ages of 1–19 years (n = 977,817 from 2012, n = 906,556 from 2013, n = 967,193 from 2014, n = 945,550 from 2015, and n = 928,025 from 2016).

This study was approved by the institutional review board of Kyungpook University in September of 2018 (IRB Number KNU2018-0141).

### Drug identification and classification

Medications were matched to their active ingredients using their nine-digit Korea Drug Code listed by the HIRA, and drug classifications were matched according to the Ministry of Food and Drug Safety (MFDS) (MFDS Internal Rule No. 68; 15 May 2015) [19].

### Polypharmacy

Polypharmacy was defined as an average of two or more active ingredients per patient per day during the one-year observation period. To evaluate long-term exposure to polypharmacy, the average number of active ingredients was calculated on a yearly basis from January 1 through December 31. In this calculation, each active ingredient in a single combination drug was counted.

### Complex chronic conditions

Complex chronic conditions (CCCs) were used to evaluate concomitant chronic diseases in pediatric patients. CCCs were categorized into nine groups: cardiovascular, respiratory, neuromuscular, renal, gastrointestinal, hematologic or immunologic, metabolic, other congenital or genetic, and malignancy [20]. Additionally, we treated three prevalent chronic diseases that require long-term medication (asthma, psychiatric disease, and diabetes) in the same manner as CCCs.

### Potential drug-drug interactions and age-related contraindicated drugs

Potential drug-drug interactions (PDDIs) and age-related contraindications were defined according to guidelines established by the Korea Institute of Drug Safety & Risk Management (KIDS) [21–23]. PDDIs and age-related contraindications defined by KIDS featured 954 drug pairs and 160 active ingredients, respectively. For analysis of PDDIs, we evaluated the list of drugs prescribed on the same date and did not consider the prescription period. In the case of age-related contraindications, acetaminophen (sustained-release formulations), cetirizine, and levocetirizine, which were contraindicated due to their formulations rather than their ingredients, were excluded from the analysis.

### Analysis

Descriptive statistics were used to demonstrate the overall prevalence of polypharmacy according to individual characteristics originating between 2012 and 2016. The associations between the presence of polypharmacy and the variables considered in this study (age, sex, insurance type, hospital admission, CCC categories, and the three other chronic diseases previously mentioned) were statistically tested via logistic regression. In these analyses, the presence of polypharmacy was the dependent variable, and the previously mentioned considered variables were the independent variables. Odds ratios and 95% confidence intervals are presented using multivariable logistic regression models. To assess the association between polypharmacy and inappropriate drug use, we analyzed the prevalence of age-related contraindications and PDDIs in the presence of polypharmacy. We performed chi-square tests to determine whether there was a significant difference in the proportion of inappropriate prescriptions between the polypharmacy and non-polypharmacy groups. We also investigated commonly prescribed drug classes in the pediatric polypharmacy group. For this analysis, the numerator of the proportion represented the sum of prescription days for each active ingredient according to the drug classification system of the MFDS. The denominator represented the sum of prescription days for all active ingredients used. Then, the proportions between the polypharmacy and non-polypharmacy groups were compared.

Stratified analysis was performed using age groups (patients aged 1–7 years, 8–13 years, and 14–19 years) due to the heterogeneity of comorbidities and prescription patterns. All analyses were performed using SAS 9.4 statistical software (SAS Inc., Cary, NC, USA).

## Results

Table 1 shows the prevalence of polypharmacy by year, demographics, and chronic conditions. In pediatric patients, the 5-year prevalence (2012–2016) of polypharmacy was 3.7%. The overall prevalence of polypharmacy decreased with age: it was 9.5% for patients between the ages of 1–7 years, 0.9% for patients between the ages of 8–13 years, and 1.1% for patients between the ages of 14–19 years. A higher prevalence of polypharmacy was observed in pediatric patients with CCCs compared to those without CCCs. Patients with CCCs involving neurologic and neuromuscular diseases showed the highest prevalence of polypharmacy. Pediatric patients with other chronic conditions or any hospital admission history also had a higher prevalence of polypharmacy when compared with those without such conditions or history of hospital admission.

**Table 1:** Prevalence of polypharmacy according to demographics and chronic conditions.

	PPS 2012–2016 (N = 4,725,141)		Age 1–7 (N = 1,498,696)		Age 8–13 (N = 1,442,468)		Age 14–19 (N = 1,783,977)	
	N	n (%)*	N	n (%)	N	n (%)	N	n (%)
**Total**	4,725,141	174,451 (3.7)	1,498,696	142,090 (9.5)	1,442,468	13,422 (0.9)	1,783,977	18,939 (1.1)
**Year**								
**2012**	977,817	35,684 (3.6)	291,335	29,484 (10.1)	317,837	2,640 (0.8)	368,645	3,560 (1.0)
**2013**	906,556	24,711 (2.7)	244,812	18,318 (7.5)	301,220	2,688 (0.9)	360,524	3,705 (1.0)
**2014**	967,193	41,316 (4.3)	323,453	34,445 (10.6)	286,009	2,843 (1.0)	357,731	4,028 (1.1)
**2015**	945,550	36,133 (3.8)	322,062	29,972 (9.3)	269,550	2,495 (0.9)	353,938	3,666 (1.0)
**2016**	928,025	36,607 (3.9)	317,034	29,871 (9.4)	267,852	2,756 (1.0)	343,139	3,980 (1.2)
**Sex**								
**Male**	2,433,231	98,707 (4.1)	772,355	78,570 (10.2)	752,262	8,907 (1.2)	908,614	11,230 (1.2)
**Female**	2,291,910	75,744 (3.3)	726,341	63,520 (8.7)	690,206	4,515 (0.7)	875,363	7,709 (0.9)
**Insurance type**								
**Health insurance**	4,580,818	168,344 (3.7)	1,476,761	140,104 (9.5)	1,399,804	12,012 (0.9)	1,704,253	16,228 (1.0)
**Medical Aid beneficiary**	144,323	6,107 (4.2)	21,935	1,986 (9.1)	42,664	1,410 (3.3)	79,724	2,711 (3.4)
**Any admission**								
**No**	4,364,348	12,3304 (2.8)	1,318,956	99,602 (7.6)	1,369,620	10,341 (0.8)	1,675,772	13,361 (0.8)
**Yes**	360,793	51,147 (14.2)	179,740	42,488 (23.6)	72,848	3,081 (4.2)	108,205	5,578 (5.2)
Complex chronic conditions (CCC)**								
**Neurologic and neuromuscular**	42,182	12,696 (30.1)	10,350	3,331 (32.2)	12,198	3,510 (28.8)	19,634	5,855 (29.8)
**Cardiovascular**	24,829	3,729 (15.0)	9,290	2,113 (22.7)	5,203	504 (9.7)	10,336	1,112 (10.8)
**Respiratory**	2,593	553 (21.3)	1,040	284 (27.3)	523	105 (20.1)	1,030	164 (15.9)
**Renal and urologic**	14,627	1,937 (13.2)	6,038	979 (16.2)	2,819	301 (10.7)	5,770	657 (11.4)
**Gastrointestinal**	29,077	2,996 (10.3)	3,479	911 (26.2)	7,018	507 (7.2)	18,580	1,578 (8.5)
**Hematologic or immunologic**	13,538	2,867 (21.2)	8,324	1,928 (23.2)	2,452	363 (14.8)	2,762	576 (20.9)
**Metabolic**	93,013	8,245 (8.9)	15,579	2,919 (18.7)	26,855	1,493 (5.6)	50,579	3,833 (7.6)
**Other congenital or genetic defect**	50,862	2,726 (5.4)	10,046	1,312 (13.1)	21,813	679 (3.1)	19,003	735 (3.9)
**Malignancy**	11,913	1,901 (16.0)	2,499	684 (19.9)	3,184	427 (7.3)	6,230	790 (7.3)
**Neonatal**	1,605	385 (24.0)	1,300	349 (26.8)	252	26 (10.3)	53	10 (18.9)
**Other**	179	27 (15.1)	33	6 (18.2)	55	7 (12.7)	91	14 (15.4)
**Any CCC**	241,332	27,560 (11.4)	58,155	11,343 (19.5)	70,749	5,555 (7.9)	112,428	10,662 (9.5)
**Other chronic conditions**								
**Psychiatric disease**	174,190	22,131 (12.7)	33,257	5,332 (16.0)	46,705	6,550 (14.0)	94,228	10,249 (10.9)
**Asthma**	1,040,724	120,639 (11.6)	683,067	112,254 (16.4)	224,920	5,050 (2.2)	132,737	3,335 (2.5)
**Diabetes Mellitus**	22,722	2,293 (10.1)	3,121	568 (18.2)	6,587	398 (6.0)	13,014	1,327 (10.2)

PPS = Pediatric Patients Sample

* Number of patients with polypharmacy and percent. Pediatric polypharmacy was defined as pediatric patients with a mean of ≥2 prescriptions on a yearly basis.

** The classification of pediatric complex chronic conditions followed criteria established in Feudtner et al. (2014).

Multivariable logistic models showed that patients between the ages of 1–7 years have a seven times greater risk of polypharmacy than patients between the ages of 14–19 years after adjusting for chronic conditions and history of admission (OR = 7.14, 95% CI = 7.02–7.26). Also, pediatric patients with CCCs had a significantly increased risk of polypharmacy. The likelihood of polypharmacy due to chronic diseases was greater in patients between the ages of 8–19 years than in patients between the ages of 1–7 years. These patterns were prominent in patients with neurologic and neuromuscular CCCs and in patients with psychiatric disease. Pediatric patients with Medical Aid benefits (OR = 1.45, 95% CI = 1.40–1.49) or any admission history (OR = 2.68, 95% CI = 2.64–2.71) had a significantly higher risk of polypharmacy (Table 2).

**Table 2:** Odds ratios for polypharmacy according to demographics and chronic conditions: PPS 2012–2016.

	PPS 2012–2016 (N = 4,725,141)	Age 1–7 (N = 1,498,696)	Age 8–13 (N = 1,442,468)	Age 14–19 (N = 1,783,977)
	OR (95% CI)	OR (95% CI)	OR (95% CI)	OR (95% CI)
Year				
2012	Ref	Ref	Ref	Ref
2013	0.82 (0.81–0.84)	1.01 (0.99–1.03)	1.12 (1.05–1.19)	1.10 (1.04–1.16)
2014	1.07 (1.06–1.09)	0.92 (0.91–0.94)	1.28 (1.21–1.36)	1.18 (1.12–1.25)
2015	0.89 (0.88–0.91)	0.78 (0.77–0.80)	1.10 (1.03–1.17)	1.02 (0.97–1.08)
2016	0.89 (0.87–0.90)	0.77 (0.75–0.78)	1.17 (1.10–1.25)	1.07 (1.01–1.12)
Sex				
Male	1.15 (1.14–1.16)	1.14 (1.12–1.15)	1.5 (1.44–1.57)	1.4 (1.35–1.45)
Female	Ref			
Age				
1–7	7.14 (7.02–7.26)	-	-	-
8–13	0.88 (0.86–0.90)	-	-	-
14–19	Ref	-	-	-
Continuous	-	0.66 (0.65–0.66)	0.91 (0.90–0.92)	0.99 (0.99–1.00)
Insurance type				
Health insurance	Ref			
Medical Aid beneficiary	1.45 (1.40–1.49)	1.05 (0.99–1.10)	2.04 (1.89–2.20)	1.99 (1.89–2.11)
Any admission				
No	Ref	Ref	Ref	Ref
Yes	2.68 (2.64–2.71)	2.22 (2.19–2.25)	2.23 (2.10–2.36)	2.22 (2.12–2.33)
Complex chronic conditions (CCC) *				
Neurologic and neuromuscular	10.12 (9.76–10.49)	3.22 (3.03–3.41)	15.15 (14.15–16.22)	15.95 (15.16–16.78)
Cardiovascular	2.19 (2.09–2.30)	1.57 (1.48–1.67)	3.87 (3.28–4.56)	2.82 (2.53–3.13)
Respiratory	1.5 (1.29–1.73)	1.58 (1.33–1.88)	2.79 (1.85–4.19)	2.39 (1.79–3.21)
Renal and urologic	1.82 (1.71–1.95)	1.51 (1.40–1.64)	2.85 (2.36–3.44)	4.53 (3.97–5.17)
Gastrointestinal	2.64 (2.49–2.79)	2.00 (1.82–2.19)	2.61 (2.26–3.02)	3.02 (2.77–3.31)
Hematologic or immunologic	1.99 (1.89–2.11)	1.35 (1.27–1.44)	5.77 (4.8–6.95)	8.97 (7.65–10.53)
Metabolic	1.84 (1.78–1.91)	1.61 (1.53–1.70)	2.31 (2.11–2.53)	2.46 (2.31–2.62)
Other congenital or genetic defect	1.06 (1.00–1.12)	1.23 (1.15–1.33)	1.52 (1.35–1.71)	1.99 (1.78–2.22)
Malignancy	3.04 (2.82–3.27)	2.64 (2.36–2.95)	4.73 (3.98–5.62)	3.69 (3.23–4.20)
Neonatal	0.55 (0.45–0.68)	0.76 (0.65–0.9)	2.00 (0.98–4.07)	0.37 (0.09–1.49)
Other	2.12 (1.15–3.92)	0.76 (0.29–2.00)	3.43 (1.07–11.03)	5.62 (2.33–13.56)
Other chronic conditions				
Psychiatric disease	5.17 (5.06–5.28)	1.89 (1.83–1.97)	18.83 (18.03–19.67)	11.54 (11.11–11.97)
Asthma	4.33 (4.28–4.38)	3.60 (3.55–3.65)	3.01 (2.89–3.14)	2.19 (2.09–2.30)
Diabetes Mellitus	1.69 (1.59–1.80)	1.36 (1.22–1.52)	1.40 (1.18–1.65)	1.76 (1.59–1.96)

PPS = Pediatric Patients Sample

OR = odds ratios; CI = confidence intervals

*The classification of pediatric complex chronic conditions followed criteria established in Feudtner et al. (2014).

**Fig 1:**
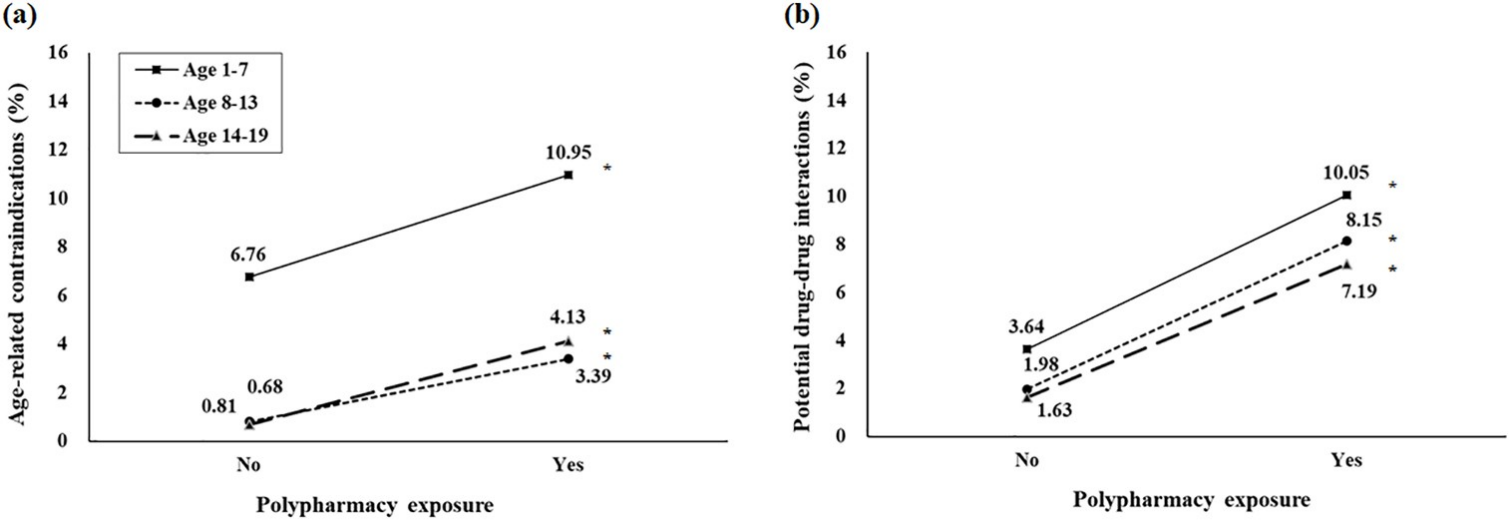
The proportions of drug-age contraindications and drug-drug interactions by polypharmacy group: (a) age-related contradictions (b) potential drug-drug interactions; * indicates a p-value <0.001 for the chi-square test.

Fig 1 shows the association between PDDIs and age-related contradictions in the presence of polypharmacy. The prevalence of inappropriate prescriptions was significantly higher for all age groups within the polypharmacy group. Among the three age groups, patients between the ages of 1–7 years showed the highest prevalence of PDDIs and age-related contradictions. Among patients between the ages of 1–7 years with polypharmacy exposure, approximately 10.1% and 11.0% had PDDIs and age-related contradictions in their medication use, respectively. The three most frequent PDDIs involved domperidone and clarithromycin, domperidone and mequitazine, and domperidone and metoclopramide. The three most frequently prescribed drugs involved in age-related contradictions were mequitazine for patients younger than 24 months, loratadine for patients younger than 6 years, and thiocolchicoside/aescin for patients younger than 16 years.

Table 3 shows the prescription dosage for each drug class in the polypharmacy group. Regardless of age, respiratory agents were the most frequently administered drugs (29%), followed by anti-allergic drugs (18.7%), central nervous system drugs (15.9%), antibiotics (10.1%), and gastrointestinal drugs (7.7%). However, the prescription pattern varied by age group. Among patients between the ages of 1–7 years with polypharmacy exposure, respiratory agents were most commonly prescribed (34.4%), followed by anti-allergic drugs (21.3%), and antibiotics (10.1%). Among patients between the ages of 8–19 years with polypharmacy exposure, the most frequently prescribed drug was a central nervous system drug that accounted for approximately half of all prescriptions (47.3% for ages 8–13 years and 49.0% for ages 14–19 years). Among central nervous system drug subclasses, antipsychotic drugs were prescribed most often (17.9% for ages 8–13 years and 22.5% for ages 14–19 years).

**Table 3:** Proportions of prescribed drugs by MDFA drug class among pediatric patients with polypharmacy.

Drug class (description)	Drug class (no.)	Total		Age 1–7		Age 8–13		Age 14–19	
		n	%	n	%	n	%	n	%
**Nervous and sensory system agents**	**100**								
Central nervous system drugs	110	28,254,509	15.9	10,044,033	7.2	6,998,920	47.3	11,211,556	49.0
Central nervous system drugs (others)	other	10,646,984	6.0	1,707,891	1.2	3,798,765	25.7	5,140,328	22.5
Antipyretics, analgesics	114	8,341,667	4.7	6,869,790	4.9	547,472	3.7	924,405	4.0
Antipsychotic drugs	117	9,265,858	5.2	1,466,352	1.0	2,652,683	17.9	5,146,823	22.5
Peripheral nervous system drugs	120	1,129,948	0.6	500,424	0.4	214,577	1.4	414,947	1.8
Sensory system drugs	130	11,902,310	6.7	10,866,100	7.8	430,213	2.9	605,997	2.6
Anti-allergic drugs	140	33,167,819	18.7	29,756,654	21.3	1,744,223	11.8	1,666,942	7.3
**Individual system agents**	**200**								
Cardiovascular system drugs	210	2,761,098	1.6	648,337	0.5	518,590	3.5	1,594,171	7.0
Respiratory system drugs	220	51,206,292	28.8	48,507,083	34.4	1,707,970	12.4	991,239	5.4
Antitussives, expectorants and mucolytic drugs	222	48,868,207	27.5	46,304,478	33.1	1,631,895	11.0	931,834	4.1
Respiratory system drugs (others)	other	2,338,085	1.3	2,202,605	1.3	76,075	1.3	59,405	1.3
Gastrointestinal tract drugs	230	13,501,522	7.7	9,565,662	7.7	1,004,310	7.7	2,931,550	7.7
Endocrine and hormonal drugs	240	3,844,977	2.2	2,449,171	1.7	512,236	3.5	883,570	3.9
Genito-urinary system and hemorrhoid drugs	250	149,628	0.1	57,346	0.0	36,467	0.2	55,815	0.2
External use drugs	260	463,140	0.3	311,533	0.2	53,745	0.4	97,862	0.4
**Metabolic agents**	**300**								
Vitamins	310	592,643	0.3	163,755	0.1	133,483	0.9	295,405	1.3
Nutrient, tonic, and alternatives	320	754,489	0.4	297,492	0.2	144,741	1.0	312,256	1.4
Blood and body fluid drugs	330	577,216	0.3	409,429	0.3	66,654	0.5	101,133	0.4
Artificial perfusion solutions	340	106,281	0.1	67,495	0.0	11,764	0.1	27,022	0.1
Metabolic drugs	390	5,859,144	3.3	4,853,022	3.3	302,935	3.3	703,187	3.3
**Agents for functional activation of organic cell**	**400**	121,305	0.1	56,428	0.0	25,810	0.2	39,067	0.2
**Biological agents against pathogenic bacteria**	**600**								
Antibiotic preparations	610	17,608,726	10.1	16,487,709	10.1	590,360	10.1	530,657	10.1
Chemotherapy for pathogenic bacteria	620	709,509	0.4	500,573	0.4	60,926	0.4	148,010	0.6
Biological preparations and miscellaneous	630	5,716	0.0	2,545	0.0	1,142	0.0	2,029	0.0
Parasite agents	640	107,492	0.1	4,697	0.0	15,104	0.1	87,691	0.4
**Opioids**	**800**	4,822,081	2.7	4,421,155	3.2	232,938	1.6	167,988	0.7
**Total number of prescriptions**		177,658,311		139,974,834		14,809,630		22,873,847	
**Number of prescriptions for which the drug class was not defined**		252		252		0		0	

**MFDS = Ministry of Food and Drug Safety**

We also analyzed the prevalence of polypharmacy based on different calculation methods. We calculated the proportion of patients with a maximum number of prescribed drugs (0, 1–4, 5–9, and ≥ 10) for at least one day during the one-year observation period in 2016. The proportion of each exposure level was 2.9%, 16.5%, 71.9%, and 8.7%, respectively. This indicates that 80.6% of pediatric patients were prescribed more than five drugs concurrently on at least one day during the 1 year follow-up period (92.5% for ages 1–7 years, 76.3% for ages 8–13 years, and 72.9% for ages 14–19 years) (S1 Table).

## Discussion

There have been inconsistencies in the definition of polypharmacy. Some studies qualified polypharmacy using terms such as duplication, drug-drug interaction, and non-match between medication and diagnosis, which confer a clinical meaning regarding inappropriate medication. However, most studies examining polypharmacy use the text definition that is based on the number of medications prescribed [2, 24]. The most commonly referenced number was more than 5 drugs for adults [2, 24] and more than 2 drugs for children [14]. In addition, the method for measuring polypharmacy exposure that considers the number of concurrent drugs prescribed is not standardized. The measurement that uses the maximum number of concurrently prescribed drugs per day, which is the most frequently used method, can provide intuitive information on the number of drugs used concurrently, but it is difficult to quantify the duration of medication. For example, children who were prescribed two drugs for only one day for one year would be considered exposed to polypharmacy [13]. Meanwhile, the calculation that relies on the daily average number of drugs prescribed has the advantage of providing both the number of medications prescribed and their duration. In this study, polypharmacy was defined and quantified as two or more active ingredients prescribed per day based on the daily average number of medications prescribed each year. Also, this study focused on describing the long-term exposure of pediatric polypharmacy. Therefore, the daily average number of drugs prescribed was used to determine polypharmacy. This method is similar to the one described in the World Health Organization (WHO) guidelines on drug use indicators, stating that an average number of medications per prescription or patient greater than two is considered polypharmacy [25].

Our results show a higher prevalence of pediatric polypharmacy and age-related differences when compared to results from previous U.S. studies. Feinstein et al. graded pediatric polypharmacy exposure based on the maximum number of concurrent drugs prescribed (no medication, 0, 2–4, 5–9, and ≥10 drugs), and the proportion of each exposure level was 45.3%, 19.5%, 27.6%, 6.6%, and 1.0% of total samples, respectively [13]. However, our results were based on a daily average of ≥2 medications during the one-year observation period, making direct comparisons with previous results difficult. Thus, to compare the results, we estimated the proportions of patients with a maximum number of prescription drugs for at least one day during the one-year observation period (0, 1–4, 5–9, and ≥ 10) using the same method as Feinstein et al. In summary, the proportions of patients with a maximum number of concurrent drugs ≥5 were 80.6% in our results and 7.6% in the U.S. study's results. This indicates that pediatric polypharmacy exposure was more prevalent in South Korea than in the U.S. Despite the relatively high prevalence of polypharmacy, the prevalence of CCCs (5.1%) in South Korea was similar to that in the U.S. (5.9%) [12]. The rate of polypharmacy was higher in patients with CCCs or a hospital admission history than those without them, which was also in line with results from the previous U.S. study. However, age-related patterns of polypharmacy were different when compared to the previous U.S. study [12, 13]. Our results showed that polypharmacy prevalence was lower in patients between the ages of 14–19 years, whereas in the U.S. study, the prevalence was higher for the same age group [13].

Comparisons between nations regarding the prevalence of polypharmacy are difficult because health problems can vary between populations and comparable data is scarce. However, since the prevalence of chronic complex diseases was similar between Korea and the U.S., it is unlikely that differences in disease severity were associated with differences in the prevalence of polypharmacy. The greater utilization of healthcare in Korea might explain the relatively high polypharmacy prevalence and its different age-related patterns among pediatric patients. Korea reported the highest number of doctor visits per person (16.0 in 2015) among the 32 OECD countries (the average for OECD countries was 6.9 in 2015) [26]. In this context, the number of office visits per person was more than two times higher in patients between the ages of 1–4 years (27.4 per year) than in patients between the ages of 5–19 years (6.6–14.5 per year) in Korea [27]. However, among U.S. patients between the ages of 1–4 years (2.8 per year) and 5–14 years (2.0 per year), the values were nearly equivalent [28]. Thus, greater utilization of healthcare may have influenced the relatively high rates of polypharmacy, especially for younger children in Korea.

We found that the inappropriate prescription of drugs, including PDDIs and age-related contradictions, increased in pediatric patients with polypharmacy, which was consistent with previous studies [13, 29]. Moreover, the proportion of inappropriate drug prescriptions was notably highest in patients between the ages of 1–7 years among the three age groups. When considering the pharmacokinetic factors of pediatric patients, adverse drug reactions are greater in younger children. During the first decade of life, age-dependent changes in body composition and organ function are dynamic, and this leads to unpredictable pharmacokinetics [30]. For example, children have more body water than body fat when compared to adults, which affects the distribution of drugs. Drug metabolic enzymes such as cytochrome P-450(CYP) isoforms and a glucuronosyltransferase (UGT) isoform are diminished in children. Thus, drugs are eliminated more slowly from the body, and drug concentrations may be increased. In addition, the structure and function of the gastrointestinal tract and kidney display age-dependent changes, and they are immature in early life [31].

In our study, the combination of antiemetic agents and antibiotics was the most frequently listed PDDI among pediatric patients. The medication selection and direction of treatment are determined by the clinical condition of the patient. However, evaluations of antiemetic agents and antibiotics may be helpful in reducing the potential harm of polypharmacy. In fact, the misuse of antiemetic agents and antibiotics has been specifically mentioned with the development of adverse drug events in pediatric patients [32–35]. In addition, we found that antipsychotic drugs were the major medication class prescribed to patients between the ages of 8–19 years with polypharmacy exposure. Previous studies have shown that the prevalence of psychotropic polypharmacy is substantial in pediatric patients [36, 37]. A study using the U.S. National Ambulatory Medical Care Survey (2004–2007) reported that 32.2% of patients visiting medical facilities for mental disorder treatment were prescribed more than 2 classes of psychotropic medications [37]. The risk of ADEs and PPDIs increased with the number of antipsychotic medications prescribed [11, 29, 38].

Our study has several limitations. First, our overall study group did not include approximately 10% of the entire South Korean population that did not visit a clinic or hospital. Therefore, our prevalence of polypharmacy is limited to prescriptions provided to pediatric patients who visit a medical institution at least once during the one-year observation period. Second, we relied exclusively on the claim database, including prescriptions issued by the medical institute. Under this condition, we could not assess the patients' compliance in taking the prescribed medication or the use of over the counter (OTC) medications. Thus, our polypharmacy prevalence results could be overestimated if patients did not take the medication even though they received the prescription. Also, our polypharmacy prevalence results could be underestimated if patients used additional OTC drugs. Third, we did not identify the appropriate indication for each medication. However, simply taking multiple medications at the same time could increase undesirable outcomes, including potential drug-drug interactions and prescription of contraindicated drugs [39, 40]. Lastly, a patient's disease state was defined using ICD-10 codes provided by the database, and there is a possibility of uncertainties in the diagnosis.

## Conclusions

Our study showed that several pediatric patients were exposed to polypharmacy that could result in inappropriate drug use. Health professionals, including pharmacists, need to be aware of this phenomenon and take action to reduce the number of medications that patients take. To aid in this, further evidence should be gathered so that pediatric medication guidelines can be established for the treatment of acute and chronic diseases. Furthermore, a system should be established to provide professional services that reduce polypharmacy exposure and prevent further unwanted outcomes in pediatric patients.

## Supporting information

S1 Table Prevalence of polypharmacy based on the maximum number of prescribed drugs for at least one day during the one-year observation period: PPS 2016. (PDF)
