# Supplementary material for: Prescribing patterns of polypharmacy in Korean pediatric patients
Source: PLoS One. 2019 Oct 1;14(10):e0222781. doi: 10.1371/journal.pone.0222781 (PMC6773215; doi:10.1371/journal.pone.0222781)
Supplement: S1 Table — (PDF) [file pone.0222781.s001.pdf]

S1 table. Prevalence of polypharmacy based on maximum number of prescribed drugs for at least one day during one year observation period: PPS 2016.

	Maximum number of prescribed drugs							
	0		1-4		5-9		≥10	
	n	%	n	%	n	%	n	%
Total	27042	2.91	153428	16.53	667180	71.89	80375	8.66
Sex								
Male	14180	52.44	78341	51.06	339885	50.94	44070	54.83
Female	12862	47.56	75087	48.94	327295	49.06	36305	45.17
Insurance type								
Health insurance	26376	97.54	149193	97.24	648176	97.15	77388	96.28
Medical aid beneficiary	666	2.46	4235	2.76	19004	2.85	2987	3.72
Any admission								
No	26896	99.46	151212	98.56	633286	94.92	29607	36.84
Yes	146	0.54	2216	1.44	33894	5.08	50768	63.16
Complex chronic conditions (CCC) *								
Neurologic and neuromuscular	95	0.35	1227	0.80	5373	0.81	2002	2.49
Cardiovascular	81	0.30	564	0.37	3572	0.54	1497	1.86
Respiratory	8	0.03	52	0.03	289	0.04	209	0.26
Renal and urologic	16	0.06	348	0.23	2311	0.35	686	0.85
Gastrointestinal	111	0.41	699	0.46	3827	0.57	1541	1.92
Hematologic or immunologic	21	0.08	214	0.14	1796	0.27	1340	1.67
Metabolic	348	1.29	2579	1.68	14965	2.24	5982	7.44
Other congenital or genetic defect	308	1.14	1628	1.06	7589	1.14	1157	1.44
Malignancy	37	0.14	319	0.21	1440	0.22	860	1.07
Neonatal	2	0.01	30	0.02	367	0.06	160	0.20
Other	0	0.00	1	0.00	6	0.00	18	0.02
Any CCC	931	3.44	6556	4.27	35341	5.30	12037	14.98
Other chronic conditions								
Psychiatric disease	343	1.27	4409	2.87	24078	3.61	4884	6.08
Asthma	45	0.17	4695	3.06	172703	25.89	45215	56.26
Diabetes Mellitus	91	0.34	728	0.47	3443	0.52	1229	1.53

PPS=Pediatric Patients Sample

* The classification of pediatric complex chronic conditions followed criteria established in Feudtner et al. (2014).
